# Modulation of Gut Microbiome in Ecstasy/MDMA-Induced Behavioral and Biochemical Impairment in Rats and Potential of Post-Treatment with *Anacyclus pyrethrum* L. Aqueous Extract to Mitigate Adverse Effects

**DOI:** 10.3390/ijms24109086

**Published:** 2023-05-22

**Authors:** Abdelmounaim Baslam, Abdelfatah Aitbaba, Asmae Lamrani Hanchi, Zakaria Tazart, Rachida Aboufatima, Nabila Soraa, Mohamed Ait-El-Mokhtar, Samia Boussaa, Marouane Baslam, Abderrahman Chait

**Affiliations:** 1 Laboratory of Pharmacology, Neurobiology, Anthropobiology and Environment, Faculty of Sciences Semlalia, Cadi Ayyad University, Marrakesh 40000, Morocco; baslamounaim@gmail.com (A.B.);; 2 Laboratory of Microbiology, University Hospital Mohamed VI, Faculty of Medicine and Pharmacy, Cadi Ayyad University, Marrakesh 40000, Morocco; 3 Department of Food Sciences and Nutrition, Faculty of Health Sciences, University of Malta, Msida 2080, Malta; 4 Laboratory of Biological Engineering, Faculty of Sciences and Technology, Sultan Moulay Slimane University, Beni Mellal 23000, Morocco; 5 Laboratory of Biochemistry, Environment & Agri-Food URAC 36, Department of Biology, Faculty of Science and Techniques—Mohammedia, Hassan II University of Casablanca, Mohammedia 20000, Morocco; 6 ISPITS—Higher Institute of Nursing and Health Techniques, Ministry of Health and Social Protection, Rabat 10000, Morocco; 7 Laboratory of Biochemistry, Department of Applied Biological Chemistry, Faculty of Agriculture, University of Niigata, Niigata 950-2181, Japan; 8 Centre d’Agrobiotechnologie et Bioingénierie, Unité de Recherche Labellisée CNRST (Centre AgroBiotech-URL-CNRST-05), Université Cadi Ayyad, Marrakesh 40000, Morocco; 9 Laboratory of Agro-Food, Biotechnologies and Valorization of Plant Bioresources (AGROBIOVAL), Department of Biology, Faculty of Science Semlalia, Cadi Ayyad University (UCA), Marrakesh 40000, Morocco

**Keywords:** brain–gut axis, gut microbiota, MDMA, dependence, conditioned place preference, microbial composition, dysbiosis, addiction, depression/withdrawal

## Abstract

The use of illicit substances continues to pose a substantial threat to global health, affecting millions of individuals annually. Evidence suggests the existence of a ‘brain–gut axis’ as the involving connection between the central nervous system and gut microbiome (GM). Dysbiosis of the GM has been associated with the pathogenesis of various chronic diseases, including metabolic, malignant, and inflammatory conditions. However, little is currently known about the involvement of this axis in modulating the GM in response to psychoactive substances. In this study, we investigated the effect of MDMA (3,4-methylenedioxymethamphetamine, “Ecstasy”)-dependence on the behavioral and biochemical responses, and the diversity and abundance of the gut microbiome in rats post-treated (or not) with aqueous extract of *Anacyclus pyrethrum* (AEAP), which has been reported to exhibit anticonvulsant activity. The dependency was validated using the conditioned place preference (CPP) paradigm, behavioral, and biochemical tests, while the gut microbiota was identified using matrix-assisted laser desorption ionization–time of flight mass spectrometry (MALDI-TOF MS). The CPP and behavioral tests confirmed the presence of MDMA withdrawal syndrome. Interestingly, treatment with AEAP led to a compositional shift in the GM compared to the MDMA-treated rats. Specifically, the AEAP group yielded a higher relative abundance of *Lactobacillus* and *Bifidobacter*, while animals receiving MDMA had higher levels of *E. coli*. These findings suggest that *A. pyrethrum* therapy may directly modulate the gut microbiome, highlighting a potential target for regulating and treating substance use disorders.

## 1. Introduction

An estimated 284 million individuals over the age of 15 worldwide have used illegal drugs within the past year, increasing by 26% over the previous decade [1]. Despite reports of the harmful effects of drug dependence on individuals and society as a whole [2], illicit substance use remains highly prevalent and contributes significantly to the global burden of diseases. However, the factors underlying dependence and vulnerability are not yet fully understood, and effective therapies are lacking. 3,4-methylenedioxymethamphetamine (MDMA), the primary component of Ecstasy tablets, is an amphetamine derivative with pharmacological effects that can stimulate euphoric and hallucinogenic sensations [3]. MDMA, predominantly used for recreational purposes, can cause toxic effects in both the central nervous (CNS) and cardiovascular systems. The development of MDMA tolerance is a common occurrence, manifested by the increased frequency and quantity of usage as part of the body’s compensatory mechanisms. MDMA primarily releases serotonin through its interaction with the serotonin transporter (5-HT), and its continued use can lead to anxiety and depression [4,5]. In addition, MDMA effects the gamma-aminobutyric acid (GABA) and dopaminergic systems by increasing dopamine release levels and causing a chronic signal imbalance in D2-like dopamine receptors in the nucleus accumbens and mesolimbic dopaminergic system. It also affects noradrenergic transmission by causing the release of noradrenaline into the synapse through its interaction with noradrenergic nerve terminals [6]. Various psychoactive compounds, including cannabis, alcohol, nicotine, opioids, and MDMA have the ability to hijack the brain’s reward system and crucial pathways responsible for pleasurable responses, reinforcement of rewarding stimuli, as well as memory and emotional processes [7,8]. Ongoing research is imperative to explore innovative approaches that aid in the prevention, prediction, and treatment of substance abuse at various stages. A critical challenge is to develop effective therapeutic strategies that can be clinically employed to manage substance use disorders (SUDs) and mitigate withdrawal symptoms. Even after prolonged periods of abstinence, a significant number of individuals with SUDs experience relapse, highlighting the need for potent interventions to address the impact of substance abuse on the brain [9].

Recent studies have linked the gut microbiota (GM), a bacterial community residing in the gastrointestinal (GI) tract, to health and disease by modulating epigenetic, metabolomic, immune, and CNS mechanisms through dynamic bidirectional communication along the ’microbiome–gut–brain axis’ [10]. The microbes present in the GI tract perform essential functions, such as maintaining the local environment, carrying out metabolic activities, and aiding the immune response. The gut microbiota can communicate with the CNS through various pathways, including endocrine, immune, metabolic, and neuronal pathways. They produce signaling molecules that act locally, activate neuronal projections to the brain, and enter the bloodstream to be distributed throughout the body (for review, please refer to [11,12,13]). Both (pre)clinical studies and animal models have demonstrated a link between the GM and illicit substance use [14,15,16]. Studies on rats treated with cocaine and methamphetamine have revealed a connection among gut microbial depletion, reduced short-chain fatty acid levels, and an increase in cocaine and methamphetamine conditioned place preference (CPP) [17,18,19]. These findings have opened new avenues connecting the change of microbial communities—in terms of quantity, abundance, and metabolites—to neuropsychiatric disorders in patients with autism, Parkinson’s disease, schizophrenia, cognitive deficits, and substance addiction [20]. There is mounting evidence linking substance abuse to the microbiome–gut–brain axis, as well as co-occurring conditions such as stress, anxiety, and depression, which are known risk factors for addiction. These factors have a direct impact on the gut microbiome, highlighting the intricate relationship between the microbiome and substance abuse [13]. Although differences in microbial community composition within subpopulations, including humans, may influence individual variations in behavior, perceived susceptibility to disease, and symptom presentation, the potential for microbial profiles to predict vulnerability to addiction has yet to be explored.

The gut microbiome plays a significant role in maintaining mental balance, which can be modulated through various approaches, including dietary patterns, probiotics, and prebiotics. Scientific studies have led to the emergence of the term “psychobiotic revolution”, which highlights the potential mental health benefits associated with microorganisms. Medicinal plants are promising candidates for psychobiotic interventions because of their diverse mix of constituents that can interact with the gut microbiota, establishing a bidirectional relationship. By targeting the microbiome–gut–brain axis, medicinal plants have the potential to exert positive effects on mental health, but further research is required to fully explore and harness their potential as psychobiotic candidates [21]. The gut microbiome has the ability to metabolize various plant compounds, including those found in medicinal plants, generating metabolites with distinct pharmacological profiles and bioavailability. These compounds can also modify the composition and diversity of the gut microbiome, promoting the growth of beneficial bacteria that contribute to overall health. As a result, the concept of ‘phyto-psychobiotics’ has emerged, emphasizing the positive effects of medicinal plants on brain health through their interactions with the microbiome. These effects are often attributed to the anti-inflammatory properties mediated by microbial metabolites derived from the active secondary compounds present in plants [22], or even by antibiotic-like effects by reducing the level of pathogenic bacteria [23,24].

*Anacyclus pyrethrum* L. (Asteraceae) is commonly used for its neuropharmacological effects in treating various diseases, owing to its GABA simulative function and increased glutamatergic neurotransmission via dopamine rise in the synaptic cleft [25]. It has been reported to possess anti-inflammatory, antioxidant, immunostimulating, and anti-mutagenic activity [26,27,28]. To date, there is insufficient evidence from mechanistic animal studies to derive causality between a specific medicinal plant and microbially mediated brain function.

In this research study, we employed biochemical techniques and matrix-assisted laser desorption ionization–time of flight mass spectrometry (MALDI-TOF MS) to investigate variations in the composition and diversity of gut microbial communities between MDMA dependence-prone rats and healthy adult rats. Additionally, we investigated the potential impact of administering a medicinal plant extract to the via gavage on their gut microbial communities. Our hypothesis suggested a link between illicit substance use and microbiome composition, implying that the microbiota’s composition could influence susceptibility to illicit substance use and alter microbiome profiles. Furthermore, we postulated that post-treatment with *A. pyrethrum* could promote the growth of specific bacterial species associated with reduced vulnerability to MDMA, thus offering a potential therapeutic defense against illicit substance dependence. These post-treatment interventions hold promise for influencing drug-seeking behavior and offering potential avenues for treating illicit substances. To our knowledge, this is the first study reporting that microbiome profiles reflect MDMA intake and that the gut microbiome could aid in identifying diagnostic biomarkers for illicit substance vulnerability and predicting risk factors.

## 2. Results

### 2.1. MDMA-Induced CPP

**Figure 1:**
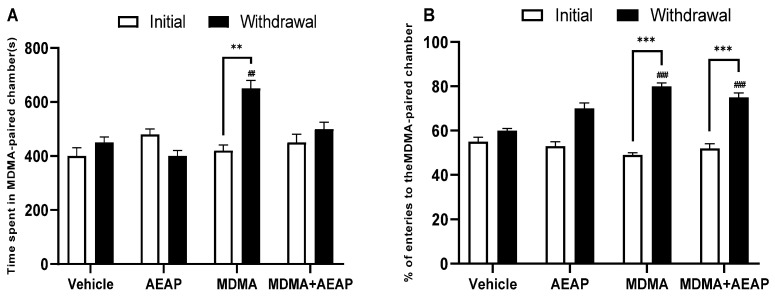
Effects of MDMA (3,4-methylenedioxymethamphetamine, “Ecstasy”) and/or aqueous extract of *Anacyclus pyrethrum* (AEAP) on dependence of conditioned place preference (CPP); (**A**) time spent and (**B**) % of entries to the MDMA-paired chamber during the CPP test. Data represent mean ± SEM (*n* = 6 per group). Student’s *t* test was used to determine whether differences existed between the two phases of each treatment ** *p* < 0.01, *** *p* < 0.001. The data on each phase (initial or withdrawal) were analyzed by one-way ANOVA followed by Tukey’s test, ^##^ *p* < 0.01, ^###^ *p* < 0.001 (compared to the vehicle control group).

The MDMA dependence was assessed using the conditioned place preference during the pre-conditioning phase. No difference was observed in time spent and % of entries to the MDMA-paired chamber among groups (Figure 1A,B). Daily MDMA administrations significantly increased the time spent and % of entries to the MDMA-paired chamber compared to the vehicle (*p* < 0.01) and preconditioning phase (*p* < 0.01), resulting in a significant establishment of CPP to MDMA. Rats spent 650 s of their time in the MDMA-paired chamber. The MDMA-dependent withdrawn group treated with AEAP showed a decrease in the time spent (500 s) and % of entries to the MDMA-paired chamber compared to MDMA-dependent rats (Figure 1A,B).

### 2.2. MDMA-Induced Behavioral Impairments and Potential of AEAP Post-Treatement to Mitigate Adverse Effects

**Figure 2:**
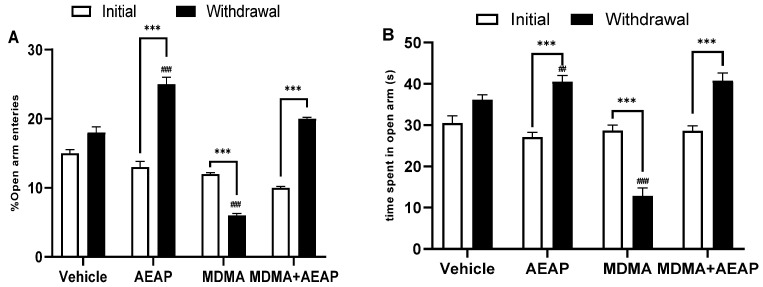
Behavioral performance on the EPM across trials under MDMA (3,4-methylenedioxymethamphetamine, “Ecstasy”) and/or aqueous extract of *Anacyclus pyrethrum* (AEAP). (**A**) % of open arms entries and (**B**) time spent in open arm entries across four trials (vehicle, rats treated with AEAP, MDMA, or MDMA + AEAP) conducted under initial and withdrawal conditions. Data represent mean ± SEM (*n* = 6 per group). Student’s *t* test was used to determine whether differences existed between the two phases of each treatment. *** *p* < 0.001. The data on each phase (initial or withdrawal) were analyzed by one-way ANOVA followed by Tukey’s test, ^##^ *p* < 0.01, ^###^ *p* < 0.001 (compared to the vehicle control group).

To elucidate the anxiety-like and depression-like behaviors, we investigated the time spent and the number of visits (% entries) to the open arms (Figure 2). During the initial phase, the results showed that there were no significant differences across the groups for elevated plus maze (EPM) and % of entries to the open arms, regardless of the treatments applied. In contrast, during the withdrawal phase, the time spent and % entries to open arms significantly increased among the AEAP group vs. the vehicle group (*p* < 0.01) and initial phase (*p* < 0.001) (Figure 2A,B). Similarly, both parameters increased in the MDMA-withdrawn group treated with AEAP vs. the initial group (*p* < 0.001), while there were no significant differences compared to the vehicle group, expressing thereby the anxiolytic effect of AEAP. The MDMA group significantly (*p* < 0.001) reduced time and entries to the open arms compared to the other treatments, revealing anxiety-like behavior during withdrawal (Figure 2A,B).

**Figure 3:**
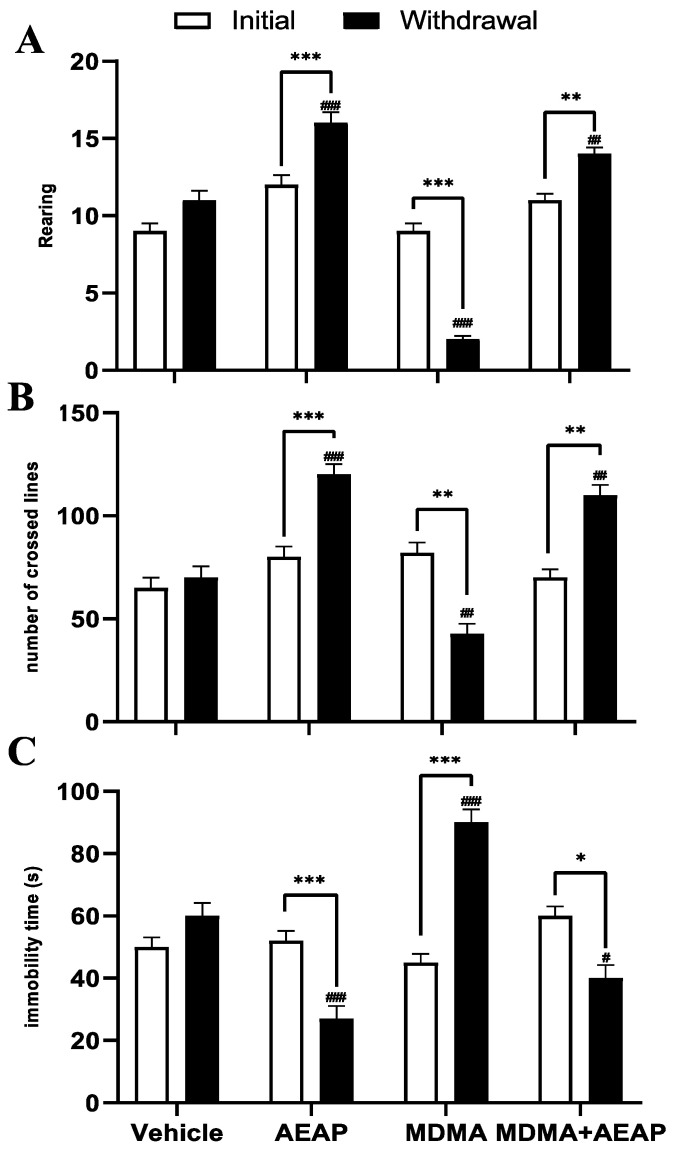
Effects of MDMA (3,4-methylenedioxymethamphetamine, “Ecstasy”) and/or aqueous extract of *Anacyclus pyrethrum* (AEAP) on the (**A**) number of rearing, (**B**) number of crossed lines in OFT, and (**C**) immobility time in FST across four trials (vehicle, rats treated with AEAP, MDMA, or MDMA + AEAP) conducted under initial and withdrawal conditions. Data represent mean ± SEM (*n* = 6 per group). Student’s *t* test was used to determine whether differences existed between the two phases of each treatment. * *p* < 0.05, ** *p* < 0.01, *** *p* < 0.001. The data on each phase (initial or withdrawal) were analyzed by one-way ANOVA followed by Tukey’s test, ^#^ *p* < 0.05, ^##^ *p* < 0.01, ^###^ *p* < 0.001 (Compared to the vehicle control group).

Figure 3 demonstrates that the number of rearings (Figure 3A) and the number of crossed lines (Figure 3B) significantly increased in rats exposed to AEAP or MDMA and treated with AEAP during the withdrawal phase compared to the vehicle group (*p* < 0.01) and the initial phase (*p* < 0.01). These characteristics were reversed in the MDMA-exposed rats. The number of rearings (*p* < 0.001; Figure 3A) and the number of lines crossed (*p* < 0.01; Figure 3B) in the open field test (OFT) significantly decreased in the MDMA group compared to the normal control rats. In the forced swim test (FST), which was used to assess depression-like behavior (Figure 3C), ANOVA showed significant differences in the immobility time among various groups. Data showed a significant decrease in immobility time in AEAP and MDMA + AEAP-treated groups (*p* < 0.01) compared to control rats and the initial phase. However, the MDMA-withdrawn group experienced increased immobility time as compared to the initial period (*p* < 0.001) and control rats (*p* < 0.001).

### 2.3. Ameliorative Effects of AEAP on MDMA-Induced Stress

**Figure 4:**
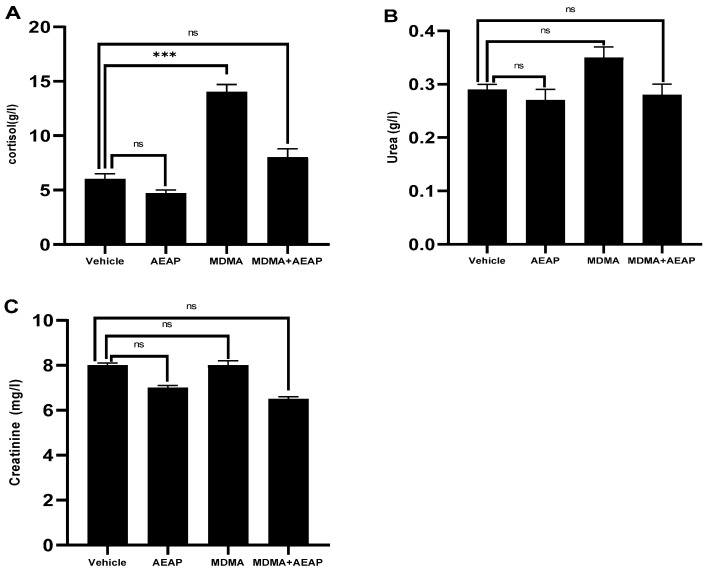
Influences of MDMA (3,4-methylenedioxymethamphetamine, “Ecstasy”) and/or aqueous extract of *Anacyclus pyrethrum* (AEAP) on the (**A**) cortisol, (**B**) urea, and (**C**) creatinine level during the withdrawal phase. Data represent mean ± SEM (*n* = 6 per group). Data were analyzed by one-way ANOVA followed by Tukey’s test, *** *p* < 0.001 (compared to the vehicle control group), ns: not significant.

The cortisol level was assessed as a biochemical marker of stress during the withdrawal phase (Figure 4A). Results showed that cortisol levels significantly increased in the MDMA-dependent group compared to vehicle (*p* < 0.001), while no difference was observed between the AEAP and MDMA groups post-treated with AEAP.

The renal function, as evaluated by measuring urea and creatinine levels, did not show any significant differences between the groups. However, the administration of AEAP, either alone or in combination with MDMA, resulted in maintaining urea levels similar to those of the vehicle group. Furthermore, AEAP administration led to a decrease in creatinine levels compared to both the MDMA-alone group and the vehicle group (Figure 4B,C).

### 2.4. AEAP Mitigates MDMA-Induced Alterations in Gut Microbiota Composition in Rats

**Figure 5:**
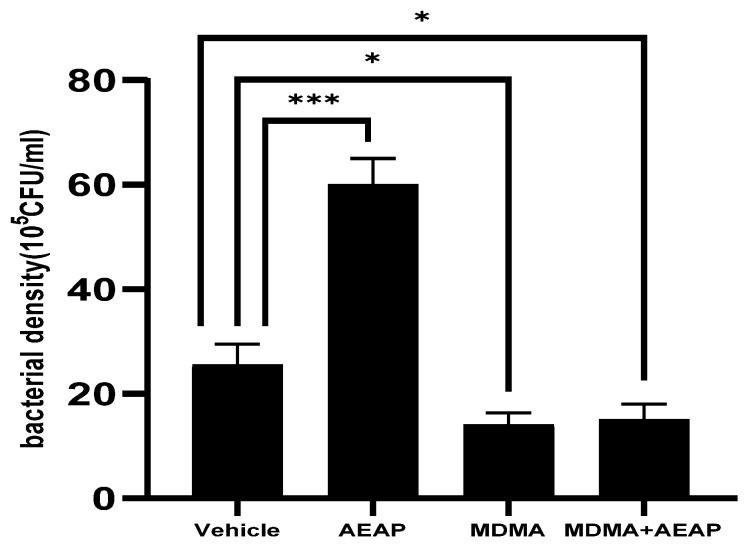
Bacterial density in control, MDMA (3,4-methylenedioxymethamphetamine, “Ecstasy”) and/or aqueous extract of *Anacyclus pyrethrum* (AEAP)-treated samples during the withdrawal phase. The density represents the total effective bacteria in the sample. Data represent mean ± SEM (*n* = 6 per group). Data were analyzed by one-way ANOVA followed by Tukey’s test, * *p* < 0.05, *** *p* < 0.001 (compared to the vehicle control group).

Bacteria from the four groups demonstrated different richness and abundance. The relative bacterial community density at the phylum and genus levels is shown in Figure 5. The AEAP group exhibited the highest bacterial density (ca. 60 × 10^5^ CFU/mL) followed by the control group (25.5 × 10^5^ CFU/mL) (*p* < 0.001). Exposure to MDMA and/or MDMA + AEAP caused disturbance of the gut microbial profile of the bacterial population, thereby displaying thereby the lowest values (15 × 10^5^ and 14 × 10^5^ CFU/mL, respectively) (Figure 5). Protein fingerprinting via matrix-assisted laser desorption ionization–time of flight mass spectrometry (MALDI-TOF MS) was employed for rapid bacterial identification in routine diagnostics and to determine the taxa that best characterized each group.

**Figure 6:**
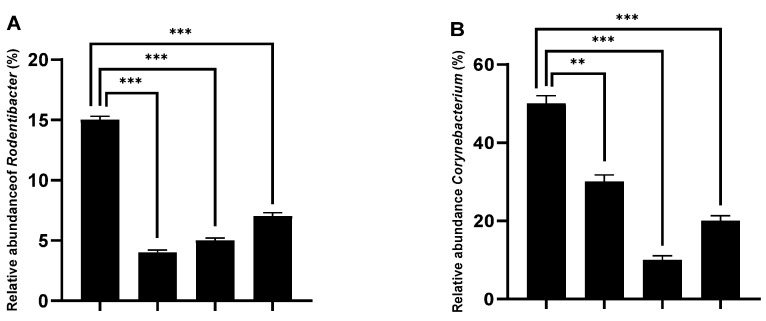

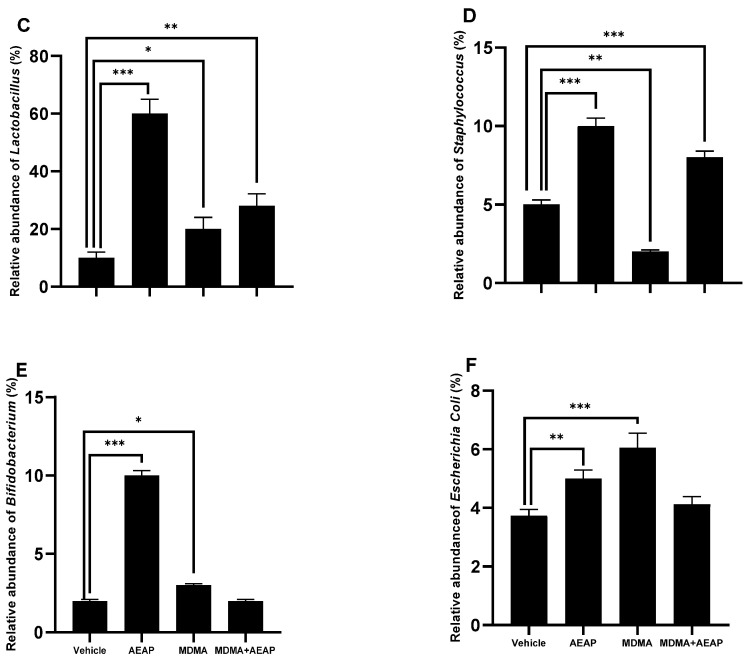
Taxonomic differences of gut microbiota among the control, 3,4-methylenedioxymethamphetamine, (MDMA)-treated, aqueous extract of *Anacyclus pyrethrum* (AEAP)-treated, and MDMA + AEAP-treated groups during the withdrawal phase. Comparison of relative abundance at the bacterial genus levels among these four groups; (**A**) *Rodentibacter*, (**B**) *Corynebacterium*, (**C**) *Lactobacillus*, (**D**) *Staphylococcus*, (**E**) *Bifidobacterium*, and (**F**) *Escherichia coli.* Data represent mean ± SEM (*n* = 6 per group). Data were analyzed by one-way ANOVA followed by Tukey’s test, * *p* < 0.05, ** *p* < 0.01, *** *p* < 0.001 (compared to the vehicle control group).

Data revealed distinct taxa in the microbiome of the control group vs. the AEAP- and/or MDMA-treated groups (Figure 6). *Rodentibacter* (ca. 15% of abundance) and *Corynebacterium* (ca. 50% of abundance) were the representative phylotypes in the vehicle control group (Figure 6A,B). The AEAP and MDMA groups exhibited the lowest values of *Rodentibacter* (4% and 5%, respectively) compared to the control group (*p* < 0.001). *Corynebacterium* represented 30% of the relative abundance for the AEAP group (*p* < 0.01), 10% for the MDMA group (*p* < 0.001), and 20% for the MDMA-dependent group post-treated with AEAP (Figure 6B). *Lactobacillus*, *Staphylococcus*, and *Bifidobacterium*, which were abundant in the AEAP group (60%, 10%, and 10%, respectively), were the key phylotypes that contributed to the difference in the gut microbiota composition between the AEAP and control groups (Figure 6C–E). MDMA-treated rats displayed a dysbiosis of the bacterial abundance profile by significantly decreasing in *Staphylococcus* (ca. 2%) and *Corynebacterium* (ca. 10%) abundance vs. the other groups. By contrast, *Escherichia coli* increased significantly in the MDMA group (ca. 6%) compared to the control (*p* < 0.001) (Figure 6F).

## 3. Discussion

The aim of this research was to investigate the behavioral, physiological, and gut microbiome responses to chronic administration of MDMA and/or the potential therapeutic effects of the aqueous extract of *A. pyrethrum* in rats. We observed perceptual changes induced by MDMA, including alterations in behavior and physiology. Previous studies have shown that MDMA can cause anxiety- and depression-like symptoms in rats, making it a reliable model for studying behavioral and neurobiological changes in humans [29]. Our data revealed that rats exposed to MDMA for 30 consecutive days showed a significant increase in the time spent and percentage of entries in the MDMA-paired chamber in the conditioned place preference (CPP) test, indicating increased preference for the illicit substance. They also exhibited increased immobility time in the forced swim test (FST), a test used to measure depressive-like behavior. In addition, rats exposed to MDMA showed an increase in the number of line crossings and number of rearings in the open field test (OFT), indicative of risk-taking behavior and impulsivity, which are common symptoms of depression [22,30,31]. The rewarding effects in MDMA-exposed rats observed in the CPP test are associated, at least in part, with dopamine release, a neurotransmitter involved in reward and pleasure [32]. MDMA enhances dopamine release and its metabolites by binding to dopamine transporters in rats [33,34]. However, excessive dopamine and dopamine turnover can have detrimental effects, and an increase in extracellular dopamine is correlated with the psychostimulant effects of MDMA [35]. In addition, the FST was conducted to assess the potential antidepressant effects of illicit drug use, as it is a reliable animal model with high predictive accuracy [36,37]. Our study found that rats exposed to MDMA showed increased immobility time during the FST, consistent with previous research documenting despair-like behavior in MDMA-exposed rats [32,38]. We investigated whether the increased immobility in the FST induced by MDMA was influenced by locomotor activity or the administration of medicinal plants with antidepressant-like effects. Therefore, we used the OFT to assess locomotor and exploratory behaviors. MDMA-exposed rats showed significant impairment in the number of rearing and crossing behaviors in the OFT.

Our study found that administering AEAP (200 mg/kg) to rats exposed to MDMA significantly reduced the CPP index and effectively mitigated depressive-like behavior, as evidenced by alterations in the increased immobility time induced by MDMA in the FST. AEAP treatment also restored the locomotor activity and exploratory behavior of MDMA-exposed rats, as shown by a significant increase in line crossings and rearing in the OFT compared to control levels. These results were consistent with previous studies that have reported the antidepressant-like and anti-epileptic potential of AEAP, which may be attributed to its anti-inflammatory, antioxidant, immunostimulating, anti-mutagenic, and locomotor-stimulant properties observed in other animal models of depression [26,27,28,39]. Therefore, our study confirmed the antidepressant-like activity of AEAP in the MDMA animal model and supported its potential therapeutic use. *A. pyrethrum* has been commonly used as a brain tonic in complementary and alternative medicine [40]. While AEAP may cause mild and transient side effects such as nausea, abdominal pain, and vomiting, the severity of these effects may vary based on factors such as dosage, duration of use, and individual factors such as age and pre-existing medical conditions [41,42,43]. It is worth noting that other studies have reported no significant side effects or adverse events associated with AEAP use [44,45].

Previous studies have shown that individuals with severe depression, inflammation-related conditions, and neurotransmitter dysregulation may exhibit elevated cortisol levels and kidney damage in response to MDMA consumption. Hence, we aimed to assess the levels of cortisol, urea, and creatinine in rats exposed to MDMA and/or treated with AEAP. Our study revealed that administering MDMA increased the levels of the neurohormone cortisol in rats but did not have a significant effect on renal function. These results were consistent with previous studies on the chronic use of various drugs of abuse, including MDMA (for a review, see [46,47,48,49,50]). Research has shown that cocaine use can also lead to elevated cortisol levels, with dependent individuals often experiencing increased stress perception and exhibiting deficits in learning and memory, which can be attributed to heightened cortisol levels. Furthermore, individuals with impaired learning and memory abilities accompanied by higher cortisol levels tend to increase their cocaine use after treatment, indicating a relationship between chronic drug use, elevated cortisol levels, impaired memory, and treatment outcomes [50]. In addition, clinical studies have demonstrated significantly elevated levels of pro-inflammatory markers in individuals with depression [51].

To further investigate the pharmacological mechanisms of AEAP, we examined whether post-treatment with AEAP could reverse the biochemical changes induced by MDMA exposure. Remarkably, we observed a significant decrease in cortisol levels following treatment with AEAP alone or in combination with MDMA in the treated animals. Altogether, this is the first report demonstrating the ability of AEAP to ameliorate the behavioral and biochemical markers, including cortisol release, in MDMA users. However, the mechanism(s) underlying the restorative effect of AEAP on MDMA-induced depression-like behavior and altered cortisol release and neurohormone levels were not elucidated. It is possible that AEAP modulates the metabolism of relevant neurotransmitters through alterations of brain-derived neurotrophic factors and/or brain development and function dependent on the diet. Notably, substances of misuse modulate dopamine release and its receptors (e.g., D2-like dopamine) [7,52] and generate, ‘hijack’, and amplify the dopaminergic appetitive system, leading to the activation of the meso-limbic pathway responsible for the pleasure and reward response, as well as memory and emotional processes [53]. Other neurotransmitters, including serotonin and GABA, play a crucial role in patients with depressive disorders [54].

The gut microbiota has been shown to regulate several host neurotransmitters, including GABA, serotonin, and dopamine [55,56]. Given the explosion of research focused on the microbiome–gut–brain axis, accumulating evidence supports the hypothesis that the gut microbiota plays a crucial role in CNS (dys)function, and there is keen interest in the development of potential and alternative therapies to improve patient outcomes with substance use disorders. Hence, we sought to determine whether administration of MDMA leads to alterations in the intestinal microbiota and examine the abilities of AEAP to attenuate responses to MDMA-induced withdrawal syndrome in rats. Our study revealed significant differences in bacterial density, specific bacterial taxa, and propionate levels in the GM of rats exposed to MDMA and/or AEAP. While previous research has explored gut microbiota differences in substance users [9,18,19], this is the first study to investigate the effect of a plant extract on the gut microbiome in an MDMA-induced animal model. We found that the GM diversity was greatest in AEAP, AEAP + MDMA, and MDMA rats in descending order, compared to healthy controls. Although greater bacterial diversity has a potential benefit to human health, its role in CNS function remains unclear. Previous reports have demonstrated an increase in both GM diversity and richness in patients with major depressive disorder [57], as well as in a group of children with autism [58]. As a result, it is uncertain how increased bacterial diversity affects MDMA dependence. Dysbiosis of the GM profile has been found during exposure to environmental cues (i.e., imbalanced diets, toxins, drugs, and pathogens) [59], and it has been associated with array of diseases, including CNS-related disorders [59], inflammatory bowel disease [60], colorectal cancer, obesity [61,62], and diabetes [63,64]. Therefore, further research is needed to explore the mechanisms underlying the observed changes in the GM and their potential implications for the development and treatment of substance use disorders.

Interestingly, it was noted that rats treated with AEAP post-MDMA exhibited a growth of *E. coli* and a repression of *Rodentibacter*, *Staphylococcus*, and *Corynebacterium* species, while untreated and AEAP-treated animals did not yield *E. coli* bacteria. This indicated that MDMA altered the abundance bacteria in the GM population differentially. These findings implied that MDMA may play a role in modulating changes in GM composition and that psychoactive substances that alter GM can contribute to the central infiltration and smooth the occurrence of substance use disorders. In contrast, administration of AEAP showed higher relative abundances of *Lactobacillus*, *Rodentibacter*, *Staphylococcus*, and *Corynebacterium* than MDMA-treated animals, which could improve gut health by reducing endotoxin production, increasing the conversion of primary into secondary bile acids, maintaining gut immune homeostasis, and promoting absorption [65].

The alteration in GM abundance induced by MDMA was consistent with previous reports showing that cocaine-treated rats have lower microbiota diversity than the vehicle group [18,19,66]. The microbiota plays a primordial role in digestion, behavior, and metabolism and can modulate the amount of diverse neuroactive molecules in the CNS [67]. Dopamine is a critical neurotransmitter for reward-driven behaviors, and it is a precursor for other catecholamines that regulate peripheral immune responses and are linked to various autoimmune diseases and neurological disorders [68]. The GM reacts to these catecholamines, and many neurotransmitters can be generated by the GM, including catecholamines (dopamine and norepinephrine), GABA, and serotonin [13]. In humans, more than 50% of dopamine is generated in the gut, and the GM modulates the peripheral dopamine levels. Bacterial species belonging to the genus *Staphylococcus* can synthesize dopamine through the use of staphylococcal aromatic amino acid decarboxylase [69]. Lactic acid bacteria (LAB), including those found in the genera *Lactobacillus*, *Bifidobacterium*, and *Streptococcus*, can synthesize GABA from GABA-enriched substrates (i.e., fermented foods and beverages). These bacteria utilize the enzyme glutamic acid decarboxylase for GABA production [12]. In human studies, it has been shown that *Lactobacillus brevis* and *Bifidobacterium dentium* were the most efficient GABA-producing bacteria among the 91 bacteria present in the gut [70].

GABA functions as the primary inhibitory neurotransmitter for the CNS, binding to specific receptors (GABA_A_ and GABA_B_) to exert its inhibitory effects on the immune system [71]. GABA is transported across the blood–brain barrier (BBB) via simple diffusion, transcytosis, or carrier-mediated transport [72]. Elevated GABA levels in the hippocampus and prefrontal cortex are likely downstream of the effects on the hypothalamic–pituitary–adrenal (HPA) axis, where digestion by the GM of nutrients influences GABA levels and could improve anxiety-related symptoms [73,74]. It is noteworthy that ingestion of the nonpathogenic bacteria *Lactobacillus rhamnosus* JB-1 has been shown to modulate the GABAergic system in mice and can reduce depressive- and anxiety-like behaviors in a vagus-dependent manner [75]. A recent study found that oral supplementation of *Bifidobacterium breve* NCIMB8807 pESHgadB, a strain genetically modified to produce GABA by overexpressing glutamate decarboxylase B in the gastrointestinal tract, reduced sensitivity to visceral pain in a rat model. This reinforces the idea that microbiota-mediated GABA can positively influence the host [76,77]. Additionally, bacterial genera of *E. coli* can produce dopamine and noradrenaline in large amounts [78]. Data herein revealed a higher relative abundance of *E. coli* in the MDMA group, indicating an indirect way for the microbiota to impact the reward system by producing certain metabolites (i.e., SCFAs), known to stimulate the release of dopamine, which is involved in the regulation of reward and motivation [79]. It has been shown that non-absorbable antibiotic use decreased the microbiota and increased vulnerability to the behavioral effects of cocaine [17].

Illicit substances abuse can alter the density and/or relative abundance of GM, thereby inhibiting the development of the protective microbiota by damaging the close-knit proteins. This can lead to increased permeability of the BBB [80] and the interaction of lipopolysaccharide-TLR4 in the gastrointestinal system, which can activate microglia that release inflammatory chemicals that modulate excitatory responses [81]. Gut inflammation and increased gut permeability can allow bacterial-derived antigens to pass into the bloodstream, which can trigger pro-inflammatory chemical release, stimulate glial cells, triggers apoptosis, and impair brain function and behavior [66]. The current treatments for substance abuse diseases mainly focus on prevention, but effective treatment options, such as a ketogenic diet, require prior identification and intervention.

Current evidence suggests that modulating the microbiota–gut axis could be an effective strategy against substance abuse [82]. Dietary interventions, which play a crucial role in modulating the GM and are linked to depression-related behaviors, have emerged as a promising approach to improving brain health. Herein, we investigated the potential of *A. pyrethrum* as a therapy for substance abuse by modulating the gut microbiota. While previous research has identified potential interactions between the GM and volatile or oil-containing medicinal plants traditionally used to treat gastrointestinal disorders [82,83], there has been limited systematic research on GM involvement in substance abuse. This study represents an initial attempt to establish a correlation between AEAP-MDMA administration and gut microbiota. AEAP exposure in rats enhanced the abundance of *Bifidobacterium*, *Lactobacillus*, and *Staphylococcus*. Several bacterial strains have recently been identified as “psychobiotic” actors, a novel class of psychotropics given the lower response rates observed for traditional antidepressants [84]. Moreover, as stated above, the oral ingestion of *Bifidobacterium infantis* resulted in increased tryptophan, a serotonin precursor, and GABA [84,85]. Our study provides new insights into the potential benefits of specific bacteria against behavioral and brain abnormalities in rats. Using mouse models, we previously found that AEAP possesses antinociceptive, anti-inflammatory, and antioxidant activities [41]. These effects may be due to the presence of interesting phytoconstituents, including N-isobutyldienediynamide and polysaccharides as major compounds, and several secondary metabolites (alkaloids, reducing compounds, tannins, flavonoids and coumarins), saponins, sesamin, inulin, gum, and traces of essential oil. HPLC analysis of AEAP revealed the presence of pellitorine, which is the main compound of the plant [41,86]. However, no investigation has been undertaken to reveal the impact of N-isobutyldienediynamide-containing plants on the microbiome–gut–brain axis, and this avenue needs further exploration. Of interest, previous studies in mice have shown that polysaccharides have beneficial effects against ulcerative colitis [87] and stress-induced depression behaviors [88] by restoring the gut microbial profile. The gut microbiota is capable of metabolizing plant-derived polysaccharides into SCFAs, which act on the gut–brain axis through (i) the neural pathway by reducing cortisol; (ii) the immune pathway by lowering inflammatory mediator levels and microglial activation; and (iii) the humoral/metabolic pathway by enhancing serotonin synthesis, neurotrophic factors, and various gut neuropeptides [89,90,91]. Several reviews and meta-analyses have provided evidence for the clinical benefits of plant-derived phytochemicals, including secondary metabolites, polyphenols, and essential oil, as phyto-psychobiotics for the treatment of mental and behavioral disorders and studies have been conducted on their interactions with the gut microbiota [92,93,94,95].

In summary, this study demonstrated that MDMA administration can cause withdrawal syndrome and changes in the biochemical marker levels, but AEAP administration can effectively mitigate these effects and prevent alterations in the gut microbiota. These findings suggest that the gut microbiota may play a role in substance abuse and offer potential avenues for addiction treatment. The observed therapeutic effect of AEAP in MDMA-exposed animals may be associated with the modulation of specific bacterial taxa in the gut microbiota. Notably, the physical–chemical relationship between the gut microbiome and its environment is essential in regulating its composition and function. For instance, the pH of the gut environment can impact the growth and survival of microbial species and influence the activity of enzymes involved in dietary component breakdown. Additionally, physical factors such as nutrient availability, oxygen levels, and host immunity can also affect the gut microbiome. Understanding the physical–chemical relationship between the gut microbiome and its environment is crucial for developing interventions to modulate the microbiome and promote host health. Moreover, further research is needed to elucidate the molecular mechanisms and gut microbiota signaling pathways involved in MDMA-induced alterations in the gut microbiota and the effects of AEAP on MDMA-induced depression.

## 4. Materials and Methods

### 4.1. Animals

Male Sprague–Dawley rats (with an average weight of 210 ± 20 g upon arrival) were kept individually in clear cages, under controlled environmental conditions of temperature (22 ± 2 °C) and humidity (50 ± 10%) and maintained on a 12 h:12 h light/dark cycle. They were provided free access to food and water *ad libitum*. The animals were acclimatized to the laboratory conditions for seven days before the experiments. All animal procedures were performed in accordance with the EU2010/63 European Council Directive’s guidelines for the Care and Use of Laboratory Animals. Approval for animal experimentation was obtained from the Institutional Review Board of the Faculty of Sciences at Cadi Ayyad University in Marrakesh, Morocco. The approval was granted in accordance with the guidelines set forth by the Committee for the Purpose of Control and Supervision of Experiments on Animals and Animal Ethics. The protocol code for this study was CA952/05/22, and it was approved in September 2022.

Following acclimatization to laboratory conditions, the animals were divided into four groups: (1) the control group (vehicle; treated with saline solution 0.9%), (2) MDMA-dependent group, (3) AEAP treatment group, and (4) MDMA + AEAP group. The MDMA-dependent group received daily administration of MDMA for 30 days, followed by post-treatment with AEAP (200 mg/kg; per gavage) for 7 days (day 34 to 40). Each group comprised six animals.

### 4.2. Drugs Administration

Racemic 3,4-methylenedioxymethamphetamine (MDMA) was purchased from Sigma Aldrich (St Louis, MI, USA) and dissolved in saline solution. The drug was administered as a daily chronic dose for 30 days by gavage to animals based on the previous pharmacological reports [8,96] in a progressively escalating dose—mimicking the progressive increases that characterize human addiction—starting from 10 mg/kg/day to 20 mg/kg/day (20% increase/week).

### 4.3. Plant Material and Preparation of the Aqueous Extract

*Anacyclus pyrethrum* roots were collected in nearby Marrakesh, Morocco (Bin El Ouidan; 32°7′48″ latitude N/6°27′36″ longitude W) and authenticated at the Department of Pharmacognosy, Cadi Ayyad University, Marrakesh. The voucher specimen (MARK-1003) was deposited at the Department of Biology, Cadi Ayyad University, Marrakesh (Morocco). Plant extraction was performed as described before [39] with slight modifications. Briefly, crushed dried root extraction was performed for 24 h under agitation with distilled water (1 g/10 mL). The aqueous macerate was centrifuged (15 min, 1200 rpm), filtered, and the concentrated extract was lyophilized to produce the powder form. The extract was evaluated for the microbial study and there was no microbial contamination (i.e., *Escherichia coli*, *Salmonella*, *Staphylococcus aureus* or *Pseudomonas aeruginosa*) in the extract. The lyophilized dry powder was sealed in amber bottles and kept at 4 °C until its use.

Plant extract does not have any acute or subchronic toxicity effects [97]. The evaluated doses for toxicity (1000, 2000, and 5000 mg/kg) of the aqueous extract of *Anacyclus pyrethrum* were safe. After 14 days of AEAP administration, no mortality and no significant changes in body or organ weights (*p* > 0.05) were monitored. The LD50 value of AEAP was greater than 5000 mg/kg, indicating the non-toxicity of the extract [98].

The selection of the 200 mg/kg dose for the present study was based on the results of a previous study conducted within our laboratory [41,97], which showed significant efficacy and yielded informative outcomes for this particular dose.

### 4.4. Conditioned Place Preference (CPP)

MDMA-induced CPP was carried out following the previously established protocol [99], with minor modifications. The CPP system consisted of three PVC compartments, including two similar-sized large conditioning side chambers (L × W × H: 30 cm × 25 cm × 30 cm) with two colored walls (white or zebra) and different floor surfaces (parallel metal bars or stiff metal mesh) serving as somatosensory cues and one neutral middle chamber (11 cm × 25 cm × 30 cm). The CPP schedule comprised three phases: preconditioning, conditioning, and postconditioning (dependence). During the pre-conditioning phase (days 1 to 3), the animals were placed in the middle chamber with the doors removed for free access to the entire apparatus and recorded for 15 min. The time and number of entries (four legs inside the chamber) were monitored on day 3, and animals that exhibited an initial preference for one side chamber over the others were removed (biased). During the conditioning phase (days 4 to 9), each rat received alternate injections of either MDMA (ecstasy) or saline twice a day (at 10:00 a.m. and 8:00 p.m.) for 6 days. The rats were assigned to the zebra compartment for 45 min immediately after MDMA administration and to the white compartment after receiving saline injections. The control group was given vehicle injections during alternating sessions in the conditioning and postconditioning phases. In the dependence postconditioning phase (days 10 to 33), rats were re-evaluated for MDMA-induced CPP by providing them with unrestricted entry to both the white and zebra compartments for 15 min. Animals that displayed an aversive effect to Ecstasy were excluded after calculating the number of entries to the MDMA-paired chamber/total entries, which was recorded as the CPP score. The time spent by rats in each of the two compartments during the 15 min test was recorded using a camera connected to a computer via an electrical interface. At the end of the withdrawal phase (day 40), the rats were given free access to both compartments for another 15 min, and their behavior was recorded. Tukey’s *t* tests were used to determine if a significant preference for one of the compartments was established. A *p*-value less than 0.05 was considered indicative of a significant difference.

On day 40, after the withdrawal phase, behavioral tests were carried out in a sound-attenuated room between 9:00 a.m. and 3:00 p.m.. Subsequently, the animals were sacrificed for further examination of their biochemical markers and gut microbiota.

### 4.5. Behavioral Tests

#### 4.5.1. Elevated Plus Maze (EPM)

The anxiety-like behaviors of the rats were assessed using the EPM during the preconditioning phase (day 1) and at the end of the withdrawal phase (day 40) in a separate room from the housing area. The EPM was elevated 100 cm above the floor and consisted of two open arms, two enclosed arms (L × W: 50 cm × 10 cm each), and a central zone (L × W: 10 cm × 10 cm). The closed arms had walls 40 cm in height, while the open arms had no walls. The test began by positioning the rat in the center of the maze, facing the intersection, and recording the exploratory behavior in the maze for 10 min. The number of open/closed arm entries (four legs on the arm) and open/closed arm time were used as the dependent measures [100]. The EPM was cleaned with 10% ethanol after each test to minimize the possibility of introducing pheromonal cues. Tukey’s *t* tests were used to determine significant differences in open/closed arm time or entries (*p* < 0.05 was considered significant).

#### 4.5.2. Porsolt’s Forced Swim Test (FST) for Depression

FST is a behavioral model used to screen for depression-like behavior in the rat [101]. Rats were subjected individually to immobility in a transparent cylinder (21 cm in diameter × 60 cm tall) containing 40 cm of water maintained at 25 ± 1 °C and the immobility time was recorded for 10 min. Immobility was defined as the period during which the rats remained motionless in the water with no active behaviors such as jumping, diving, and swimming and making only movements to keep their head above water. Increased time of immobility refers to a depressant-like effect in the behavioral profile.

#### 4.5.3. Open Field Test (OFT)

(OFT) was conducted under bright ambient room light to evaluate locomotor activity and exploratory behavior. Each rat was placed individually in the center of a white arena (80 cm × 80 cm × 40 cm) divided into 25 equal squares and allowed to explore the unfamiliar arena for 10 min. The number of squares crossed with all four legs and the number of times the rodents reared up on their hind legs to explore were recorded as measures of locomotor activity and exploratory behavior, respectively [102]. At the end of each rat’s test, the OFT apparatus was cleaned with 10% ethanol to eliminate any potential olfactory cues.

### 4.6. Biochemical Analysis

Following the behavior tests on day 40, animals were sacrificed by decapitation, and blood was collected. Blood samples were collected into ice-cooled centrifugal tubes without using an anticoagulant, clotted for 30 min at 25 °C, and centrifuged for 15 min at 1500× *g* to collect the serum, which was stored at −20 °C for biochemical estimation.

The cortisol, urea, and creatinine levels were measured using the standard technique with a biochemical machine (Cobas 6000, Roche, Basel, Switzerland).

**Figure 7:**
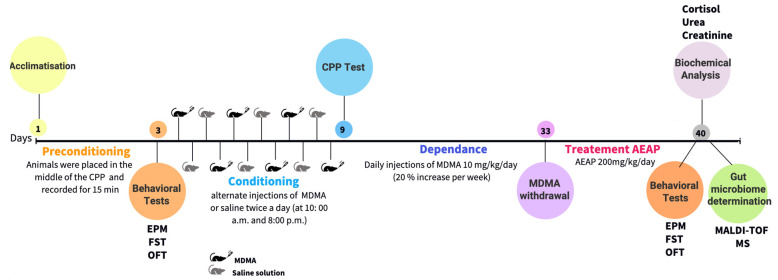
Graphical flowchart illustrating the step-by-step process of the experimental design, including the selection of rats, drug administration, behavioral tests, and sample collection for gut microbiota analysis to investigate the modulation of the gut microbiome in Ecstasy/MDMA-induced behavioral and biochemical impairment in rats and the potential of post-treatment with *Anacyclus pyrethrum* L. aqueous extract to mitigate adverse effects.

Figure 7 presents a flowchart illustrating the step-by-step process of the experimental design employed in the current study.

### 4.7. Gut Microbiota Determination

Intestinal samples were diluted with sterile physiological saline (NaCl 0.9%) and mixed thoroughly by vortexing for 15 min. Bacterial enumeration was carried out according to the surface spread method consisting of serial 10-fold dilutions (ranging from 10^−1^ to 10^−6^) of the original prepared sample. Each dilution (100 µL) was placed onto the blood agar medium. The inoculated plates were incubated under anaerobic (AnaeroGen; Oxoid, Basingstoke, UK) and aerobic conditions at 37 °C for 72 h and then the colonies were counted before MALDI-TOF MS analysis, and the results were expressed as CFUs (colony-forming units) [102]. Six strains were selected based on their significant role and representativity and were used as reference strains. Stock bacterial cultures were kept at −80 °C in 60% glycerol (Laboratory of Microbiology, Faculty of Medicine and Pharmacy, University Hospital Mohamed VI, Marrakesh, Morocco) until use.

#### 4.7.1. MALDI-TOF MS Spectra Preparation for Mass Spectral Profiles

A preparatory step involving formic acid (FA) was employed to extract the microbes before the bacterial spot was covered with the matrix solution. This was done because the acidic pH of the matrix enhances ribosomal protein extraction. In brief, the sample was mixed with 300 μL of high-pressure liquid chromatography (HPLC)-grade water, followed by the addition of 900 μL of 100% ethanol. The resulting homogenate was then centrifuged at 15,000× *g* for 2 min to obtain the bacterial pellet, which was subsequently dried and resuspended in 50 μL of FA (70% in water). The mixture was then vortexed and 50 μL of acetonitrile (ACN, Sigma, Schnelldorf, Germany) was added, followed by centrifugation at 15,000× *g* for 2 min. Next, for each strain, a bacterial extract supernatant (1 μL) was spotted in two replicates onto a polished MSP 96-spot steel plate (Bruker-Daltonics, Billerica, MA, USA) and allowed to dry at room temperature. To calibrate the instrument during acquisition and processing, a bacterial test standard (1 μL) (Bruker-Daltonics) was pipetted onto two MALDI target spots. Following this, the bacterial samples were overlaid with α-cyano-4-hydroxycinnamic acid (1 μL) matrix, air-dried, resuspended in 70% FA and CAN, and then analyzed using MALDI-TOF MS. Prior to each acquisition session, a bacterial test standard (BTS) was used for instrument calibration.

#### 4.7.2. MALDI-TOF MS Data Acquisition and Processing

The Bruker-Daltonics Microflex LT mass spectrometer was utilized to conduct MALDI-TOF MS. The protein mass spectra of the samples were obtained using the reference database V.3.1.2.0 (3995 entries), the investigation-use-only (RUO) MALDI Biotyper software (version 3.0) (Bruker-Daltonics), and a laser frequency of 20 or 60 Hz in linear and positive mode within a mass range of 2000–20,000 Da. The default operating conditions included ion source voltage at 18.25 kV, acceleration at 20 KV, and pulse ion extraction at 370 ns. Thirty single spectra were generated for each strain from four independent cultures with three technical replicates. The manufacturer’s criteria for MALDI-TOF MS analysis were used to interpret the results, where a score of 2.0 was indicative of species-level identification with high confidence, scores between 1.700 and 1.999 suggested genus-level identification, and scores below 1.7 were not assigned identities.

### 4.8. Statistical Analyses

The statistical analysis of the data was conducted using GraphPad Prism Software version 9.00 (San Diego, CA, USA). The results were presented as mean ± standard error of the mean (SEM) (*n* = 6). One-way analysis of variance (ANOVA) was performed, followed by post hoc Tukey’s tests to assess the differences between groups. A *p*-value less than 0.05 was considered statistically significant.

## 5. Conclusions

Altogether, our study shows that exposure to MDMA induced withdrawal syndrome, altered biochemical markers, and disrupted the gut microbiome. However, administration of AEAP mitigated the depressive-like behavior caused by MDMA. The observed therapeutic effect of AEAP in MDMA-exposed rats may be due, at least in part, to its ability to modulate the gut microbiota. These results highlight the gut–brain axis connection and emphasize the significance of gut health in overall wellness. Our data suggest that the gut microbiome could play a critical role in MDMA-induced SUDs and that *A. pyrethrum* extract may act as a potential ‘psychobiotic’ to modulate gut microbes and benefit mental health and associated drug-seeking behavior. These findings suggest that manipulating the gut microbiota could be a promising breakthrough therapy. However, further clinical and in vivo studies are needed to elucidate the molecular and biological mechanisms of action underlying the microbiota–gut–brain crosstalk. A deeper understanding of this axis, based on unexplored molecular and biological mechanisms, could be a great leap forward toward developing effective treatments against illicit substance dependence.

## Data Availability

Data are contained within the article.
